# Normal uniform mixture differential gene expression detection for cDNA microarrays

**DOI:** 10.1186/1471-2105-6-173

**Published:** 2005-07-12

**Authors:** Nema Dean, Adrian E Raftery

**Affiliations:** 1 Department of Statistics, University of Washington, Box 354322, Seattle, WA 98195-4322, U.S.A

## Abstract

**Background:**

One of the primary tasks in analysing gene expression data is finding genes that are differentially expressed in different samples. Multiple testing issues due to the thousands of tests run make some of the more popular methods for doing this problematic.

**Results:**

We propose a simple method, Normal Uniform Differential Gene Expression (NUDGE) detection for finding differentially expressed genes in cDNA microarrays. The method uses a simple univariate normal-uniform mixture model, in combination with new normalization methods for spread as well as mean that extend the lowess normalization of Dudoit, Yang, Callow and Speed (2002) [1]. It takes account of multiple testing, and gives probabilities of differential expression as part of its output. It can be applied to either single-slide or replicated experiments, and it is very fast. Three datasets are analyzed using NUDGE, and the results are compared to those given by other popular methods: unadjusted and Bonferroni-adjusted *t *tests, Significance Analysis of Microarrays (SAM), and Empirical Bayes for microarrays (EBarrays) with both Gamma-Gamma and Lognormal-Normal models.

**Conclusion:**

The method gives a high probability of differential expression to genes known/suspected a priori to be differentially expressed and a low probability to the others. In terms of known false positives and false negatives, the method outperforms all multiple-replicate methods except for the Gamma-Gamma EBarrays method to which it offers comparable results with the added advantages of greater simplicity, speed, fewer assumptions and applicability to the single replicate case. An R package called nudge to implement the methods in this paper will be made available soon at .

## Background

Differentially expressed genes between two or more samples may be of interest to researchers for different reasons, for example, looking at causes of or treatments for diseases such as cancer. Given appropriately processed data, the researcher needs a methodology for assessing the genes in order to separate out ones of interest, i.e. genes with "significantly" different levels of expression in different samples. Widely used methods for single slide data include examining the ratio of expression levels for the gene in each of the two samples/channels (or the log ratio), which was the quantity examined in one of the first statistical analyses for differential expression in cDNA microarrays [2]. One of the earliest uses of this quantity for determining differential expression was the "rule of two", where if the gene's ratio of expression levels in the two channels/samples is greater than two or less than half, it is considered to be differentially expressed [3].

Methods for data with replicate slides include the standard *t *test, which requires adjustment for the multiple comparisons being made. Modifications of this approach to account for multiple comparisons include the approach of Dudoit, Yang, Callow and Speed [1], which used a permutation analysis on Welsh's *t*-statistics, and the Significance Analysis of Microarrays (SAM) method, which modifies the *t*-statistic by adding a constant to the denominator [4]. A good summary of multiple testing adjustments is given by [5].

The idea of modeling the data as two groups of genes, one differentially expressed and one not, seems to be a natural and intuitive approach. This approach has been used in the context of a Bayesian analysis [6], EBarrays, assuming that the observed ratios had a gamma distribution the reciprocal of whose scale parameter itself had a gamma distribution, or, as an alternative assumption, that the observed log ratios were normally distributed and the prior for the mean was normal also. A two-component mixture model was used to model the two groups and the posterior probability was used to make inference about differential expression. This follows from work done for single slide data with a Gamma-Gamma hierarchical model [7]. Another approach using mixture models is given by Pan, Lin and Le [8].

This paper presents a very simple methodology based on mixture models called Normal Uniform Differential Gene Expression (NUDGE) detection. It is applicable to both single slide and replicated cDNA microarray datasets, produced by two of the more widely used experimental setups. After standardizing, the log ratio (or averaged across replicates log ratio) observations are modeled with a two-component mixture model; a normal component for those genes that are not differentially expressed and a uniform component for those that are. The mixture gives posterior probabilities of differential expression which do not need to be adjusted for multiple testing. This methodology is applied to three different experiments. The experiments include single replicate data (Like-Like), multiple replicate data (HIV and Apo Al), experiments with different samples being labeled with their own dyes (HIV) and experiments with all samples being labeled with one dye and compared to a reference sample (Apo Al). The results given by NUDGE are compared with those given by some other methodologies for these types of cDNA microarray experiments (different comparison methods used for different types of experiments). An R package called nudge to implement the methods in this paper will be made available soon at [9]

## Results

### HIV dataset

The HIV dataset that we analyze consists of four replicate experiments comparing cDNA from CD4+ T cell lines at 1 hour after infection with HIV-1BRU with non-infected cell lines on each slide; see [10] for details. There were four slides in total with the same RNA preparations hybridized to each. This dataset is useful in testing the specificity and sensitivity of methods for identifying differentially expressed genes, since there are 13 genes known to be differentially expressed (spots containing PCR products from segments of the HIV-1 genome which the cDNA of the infected cells should hybridize to and the non-infected should not) called positive controls, and 29 genes known not to be (non-human genes which neither infected nor non-infected cDNA samples should hybridize to) called negative controls. There are 4608 gene expression levels recorded in each replicate. The four replicates have balanced dye swaps, so no mean normalization of the (averaged across replicates) log ratios was necessary provided we always used one sample (say the infected sample) in the numerator of the log ratio and the other (non-infected sample) in the denominator regardless of which dye was used to label which sample in each array/slide.

NUDGE took a few seconds to run. All 13 positive controls, no negative controls and three other genes were found to be differentially expressed (with posterior probability greater than 0.5).

**Figure 1:**
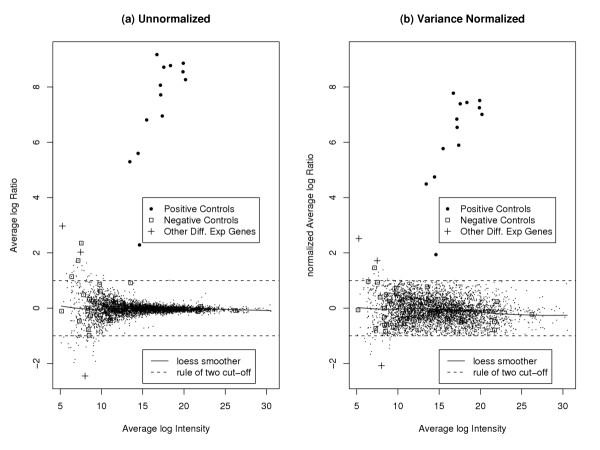
**Different Normalizations of HIV Data**. Different normalizations of HIV data: (a) raw data, (b) data normalized with respect to the variance. The bullets are the positive controls: NUDGE correctly found them all to be differentially expressed. Other genes found to be differentially expressed by NUDGE are indicated by a plus sign, and all genes found not to be differentially expressed by NUDGE are shown by small dots. Negative controls are indicated by a box. No negative controls were found to be differentially expressed by NUDGE.

It is clear from Figure 1 that the rule of two under any normalization gave less than optimal results. In all cases the rule of two correctly found the positive control genes to be differentially expressed. However, in the unnormalized case it also incorrectly found 3 of the 29 negative controls to be differentially expressed, as well as 58 other genes (including the three found by NUDGE). In the variance-normalized case, it incorrectly found one of the 29 negative controls to be differentially expressed, as well as 27 other genes (including the three found by NUDGE). Even though the rule of two is suboptimal, its performance can be improved through the use of the normalization methods suggested here.

Table 1 shows the results of different methods for the control genes. NUDGE had a perfect result for these genes, with no false positives and no false negatives. The Bonferroni-corrected *t *test was the only method considered that recorded any false negatives. The rule of two (normalized or unnormalized), SAM and the EBarrays Lognormal-Normal model all had false positives. Only the EBarrays Gamma-Gamma model equaled NUDGE's performance on these control genes.

**Table 1:** Summary of Results for HIV data for control genes

Method	Number of False Negatives	Number of False Positives
Rule of Two (on unnormalized data)	0	3
Rule of Two (on variance normalized data)	0	1
NUDGE	0	0
SAM	0	2
EBarrays (GG)	0	0
EBarrays (LNN)	0	1
*t *test	0	1
Bonferroni corrected *t *test	1	0

In order to assess the stability of the different methods, the four replicates were split into two different subsets of two replicates each (still with balanced dye swaps), and the agreements and disagreements between the genes found to be differentially expressed in each of the two datasets was calculated for each of the methods. A summary of the results is given in Table 2. The number of genes found to be differentially expressed in each of the datasets by each method is given in Table 3.

**Table 2:** Number of agreements and disagreements between the differentially expressed genes found in the two sets of two replicates for the HIV data

	NUDGE	SAM	EBarrays GG	EBarrays LNN	*t *test	Bonferroni *t *test
Agreements	14	19	13	13	34	15
Disagreements	27	153	16	32	531	217

**Table 3:** Number of genes declared to be differentially expressed by each method for the HIV data using 2 and 4 replicates

	NUDGE	SAM	EBarrays GG	EBarrays LNN	*t *test	Bonferroni *t *test
All 4 replicates	16	42	24	19	26	12
Replicates 1&3	30	49	23	27	193	83
Replicates 2&4	25	142	19	31	406	164

Comparison of results depends on how one weights agreement (roughly indicating true positives) against disagreement (roughly indicating false positives). NUDGE had more agreement and less disagreement than EBarrays-LNN, and thus dominated it on both these criteria. The *t *test, both raw and corrected, and SAM, had more agreement, but at the cost of a much higher level of disagreement than NUDGE. NUDGE had more agreement, but also significantly more disagreement, than EBarrays with a Gamma-Gamma model.

**Figure 2:**
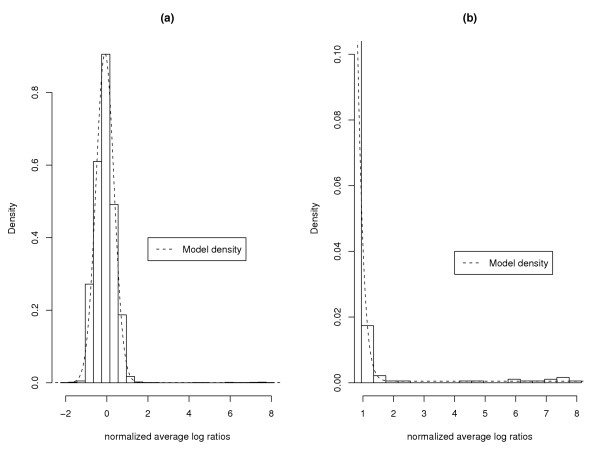
**Overlay of the model's fitted density on the normalized log ratios**. Plot (a) shows a histogram of the normalized average log ratios for the HIV data along with a dashed line showing the model-fitted density. Plot (b) shows a close-up of the right-hand tail of the histogram (where the positive controls lie) with a dashed line showing the model-fitted density.

Finally in order to check the empirical fit of the model to this data (where we know we have both differentially and non-differentially expressed genes) we plot the model's fitted density over a histogram of the normalized log ratios in Figure 2. The model seems to fit the normalized data fairly well.

### Like-like dataset

This dataset is from a microarray experiment where the same samples (with different dyes) were hybridized to an array with 7680 genes. The expression levels in the red and green dyes were extracted from the image using customized software written at the University of Washington (Spot-On Image, developed by R. E. Bumgarner and Erick Hammersmark). The genes should be equally highly expressed, as each sample is the same, so ideally we should find few differentially expressed genes.

**Figure 3:**
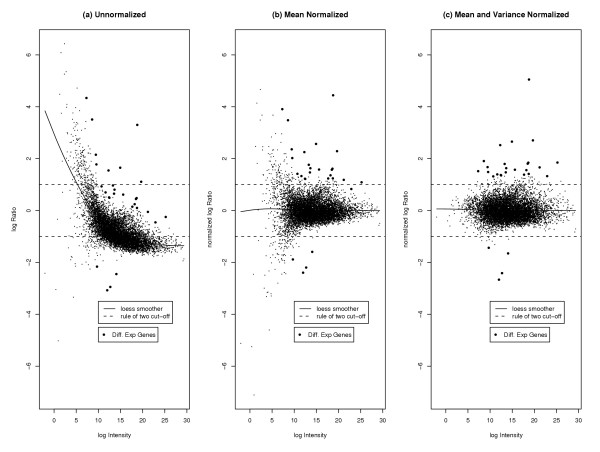
**Different Normalizations of Like-like Data**. Different normalizations of Like-like data: (a) raw data, (b) data normalized with respect to the mean, (c) data normalized with respect to both mean and variance. Diff. Exp. genes are genes found to be differentially expressed by NUDGE (with posterior probability greater than 0.5).

**Figure 4:**
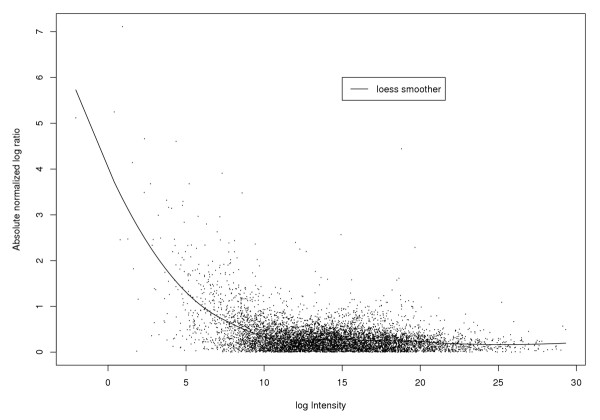
**Absolute mean normalized log ratio versus log intensity for Like-like Data**. Absolute mean normalized log ratio versus log intensity for Like-like Data. The loess line in this plot represents the estimate of the gene-specific Mean Absolute Deviation (MAD), a robust estimator of spread.

Figure 3 (a) shows the log ratios plotted against the log intensities. Here we see evidence of the dye effect, since if it were not present the data would fall with some variation about a zero-intercept horizontal line. Figure 3 (b) is a plot of the mean-normalized log ratio against the log intensity. In Figure 4 we plot the absolute mean-normalized log ratio as a function of log intensity. We use a loess smoother of this as a robust estimate of how spread depends on log intensity. This is used to get the loess variance-normalized log ratios, which are plotted against the log intensities in Figure 3 (c). The data now look much more normal and homoscedastic. The NUDGE method took less than 5 seconds to run with 10 iterations of the EM algorithm.

The results are summarized in Table 4. NUDGE found 28 differentially expressed genes (with posterior probability greater than 0.5). This is a false positive rate of 0.4%. With no normalization, the rule of two declared 3233 genes to be differentially expressed, 42.1% of the total; clearly this is not appropriate. After the data had been mean-normalized, the rule of two found 281 differentially expressed genes, a false positive rate of 3.7%. When the data have been mean- and variance-normalized, the rule of two finds 105 genes, a false positive rate of 1.4%, still higher than NUDGE. Since there is only one replicate in this case, neither *t *tests, SAM nor EBarrays can be used to test for differential expression.

**Table 4:** Results for the Like-like data

Method	Estimated False Positive Rate
Rule of Two (on unnormalized data)	42.1%
Rule of Two (on mean loess normalised data)	3.7%
Rule of Two (on mean and variance loess normalised data)	1.4%
NUDGE	0.4%

SAM, EBarrays and t tests are not applicable to single slide data.

### Apo AI dataset

This dataset was analyzed in [1] and 8 genes were suggested to be differentially expressed. The data was obtained from 8 mice with the Apo AI gene knocked out and 8 normal mice. However the replicates were not created simply by comparing samples from control labeled with one dye versus knock-out mice labeled with the other. Instead, cDNA was created from samples from each of the 16 mice (both control and knock-out) and labeled with a red dye. The green dye was used in all cases on cDNA created by pooling all 8 control mice. The statistic used in [1] was



**Figure 5:**
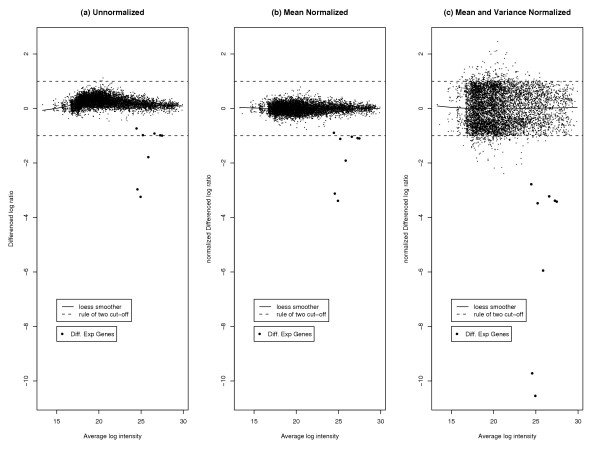
**Different Normalizations of Apo Data**. Different Normalizations of Apo data: (a) raw data, (b) data normalized with respect to the mean, (c) data normalized with respect to both mean and variance. Diff. Exp. genes are genes found to be differentially expressed by NUDGE (with posterior probability greater than 0.5).

We used the numerator of this statistic, which is the same as *M *defined in equation (7) below, in place of ordinary average log ratios, as detailed in the Methods section. Again the method took only a few seconds to run. Figure 5 shows the data at different stages of normalization along with the genes found to be differentially expressed in [1]. Table 5 shows the gene position numbers of those genes whose posterior probability of being differentially expressed was in the top sixteen found by NUDGE. All eight of the genes found by [1] to be differentially expressed were also found to be differentially expressed with high probability by our method. The lines in Figure 5 indicating the rule of two cut-off appear either to miss genes that are differentially expressed (in the unnormalized and mean-normalized cases), or to give a large number of possible false positives (in the mean- and variance-normalized case).

**Table 5:** NUDGE's Top 16 Genes from the Apo data

Top 16 genes in terms of NUDGE posterior probability of differential expression		
Row numbers in data matrix	Probability of differential expression	Found by Dudoit et al [1]?

540	1.000	Yes
2149	1.000	Yes
5356	1.000	Yes
1739	0.999	Yes
4139	0.999	Yes
2537	0.998	Yes
4941	0.993	Yes
1496	0.829	Yes

5986	0.330	No
541	0.263	No
716	0.099	No
2538	0.087	No
1224	0.066	No
799	0.060	No
1204	0.057	No
3729	0.050	No

For application of SAM, the data were normalized in the standard way, by centering the log ratios across genes within a replicate about zero. Two different levels of the SAM control parameter delta gave reasonable answers when using SAM on this data set. The first level (0.61) found 15 genes to be differentially expressed, including the eight genes found in [1] and by NUDGE, and the False Discovery Rate was estimated to be 5.3%. If we assume that only these eight genes are correct, this would actually correspond to a False Positive Rate of 46.7%. The second level (3.53) found six genes to be differentially expressed, a subset of the eight genes found by [1], and the False Discovery Rate was estimated to be 13.3%. Assuming that only those eight genes are correct, this corresponds to a False Positive Rate of 0% but a False Negative Rate of 25%. These were the best results we obtained using SAM.

For similarly normalized data, both the *t *test and the Bonferroni adjusted *t *test found the 8 genes identified by [1] to be differentially expressed. However, the *t *test found an additional 852 genes to be differentially expressed at the 5% significance level (13.5% of all genes), and the Bonferroni adjusted *t *test found an additional two genes to be differentially expressed. A summary of the results for the Apo data is given in Table 6.

**Table 6:** Results for the Apo data

Method	Number of 8 Dudoit et al [1] genes found to be differentially expressed	Number of other genes found to be differentially expressed
Rule of Two (on unnormalized data)	3	0
Rule of Two (on mean normalized data)	7	0
Rule of Two (on mean and variance normalized data)	8	134
NUDGE	8	0
SAM (delta = 0.61)	8	7
SAM (delta = 3.53)	6	0
*t *test	8	852
Bonferroni corrected *t *test	8	2

## Conclusion

We have proposed a simple method for detecting differentially expressed genes that is fast and can be applied to single-slide and multiple-replicate experiments, as well as to log ratio difference experiments. It accounts for the multiple comparisons involved, and produces a posterior probability of differential expression for each gene, rather than just a yes/no testing result. The posterior probabilities can be used either to declare which genes are differentially expressed, or to produce a ranked list of genes for further analysis. The method worked well for the three datasets that we analyzed. In terms of known false positives and false negatives, the method outperforms all multiple-replicate methods except for the Gamma-Gamma EBarrays method to which it offers comparable results with the added advantages of greater simplicity, speed, fewer assumptions and applicability to the single replicate case.

Our method can be seen as a parametric alternative to adjustment of tests for multiple comparisons using false discovery rate ideas [11], or empirical Bayes formulations [12]. A similar idea was proposed in [13] for large numbers of tests, in which the distribution of the test statistic was modeled as a mixture of two normals, one corresponding to the null hypothesis being true, and the other to its being false. This differs from our approach in that we use a uniform distribution for the mixture component that corresponds to departures from the null, rather than a mean-shifted normal. Because of this, a method such as [13] could not find both over- and underexpressed genes.

A similar idea with different distributional assumptions, using only normally distributed components is given in [8]. Instead of the average log ratios used in the method presented in this paper, [8] use a t-type statistic using the difference of average gene intensities. A more complex approach given by [14] involves modeling each level of differential expression with its own normal component.

In our approach the important aspect of the mixture is the cutoff points where the weighted normal density falls below the height of the weighted uniform density. Points beyond the cutoff are declared to be differentially expressed (under a 0.5 posterior probability rule). These cut-off points are relatively unaffected by outliers which affect the range of the data and thus the range and height of the uniform component, because the normal density falls off very rapidly towards the tails, and also because the estimated mixture weights change accordingly.

An important part of the method is normalization in terms of variance as well as mean. This extends the original lowess normalization in [1]. As a preprocessing step, it improves the performance not only of NUDGE, but also of other methods, including the simplest of all, the rule of two. Thus, this normalization method may be useful as a preprocessing tool for analysis of differential gene expression, regardless of which method is used to draw final inferences.

## Methods

### Model for detecting differential expression

Our methods are applied to averages of normalized log ratios; we discuss the specification of these quantities in different experimental settings in the section on normalizations below. In this section we will refer to them simply as observed log ratios. We use logarithms to base 2.

Our model is a normal-uniform mixture model [15,16]. We begin by modeling the genes as two different groups: differentially expressed and non-differentially expressed. Each group is modeled by its own density, and so the data as a whole are modeled by a weighted mixture of these densities, where the weights correspond to the prior probabilities of being in each of the two groups. This results in a mixture model with two components. Since genes that are not differentially expressed have a true log ratio of zero, we model the observed log ratios for these genes, after an appropriate transformation, as a group with a Gaussian density. The differentially expressed genes have log ratios that are, for the most part, in some sense "far" from the other group. So these genes can be viewed as outliers from the main distribution of non-differentially expressed genes. These genes are modeled as uniformly distributed over an appropriately wide range.

The model is



where *x*_*i *_is the observed log ratio for gene *i*, *π *is the prior probability that a gene is not differentially expressed, *N *(*x*|*μ*, *σ*^2^) denotes a Gaussian distribution with mean *μ *and variance *σ*^2^, *U*_[*a*,*b*]_(*x*) denotes a uniform distribution on the interval [*a*, *b*], and *N *is the number of genes.

We estimate the model by maximum likelihood using the EM algorithm [17]. We define the unknown labels, *z*_*i*_*, i = *1,..., *N*, where *z*_*i *_is 0 if gene *i *is not differentially expressed and 1 if it is. There are two steps in the algorithm: the Expectation, or E step, where the labels are estimated given the current parameter estimates, and the Maximization, or M step, where the model parameters, *π*, *μ *and *σ*^2^, are estimated given the current estimates of the labels. The maximum likelihood estimates of *a *and *b *are  = min{*x*_*i *_: *i *= 1,..., *N*}, and  = max{*x*_*i *_: *i *= 1,..., *N*}; these do not change during the algorithm. The steps in the algorithm are as follows:

#### Iteration k

##### Expectation Step



##### Maximization Step



The likelihood for the model given parameter estimates at iteration *k *is



The above steps are iterated until convergence. Convergence can be checked by calculating the parameter estimates, the labels, and the logarithm of the likelihood at each step, given the current estimates of the parameters. Once the change in these quantities between steps gets small enough the algorithm is deemed to have converged. The increasing property of the EM algorithm guarantees that a local maximum is reached, but a global maximum cannot be guaranteed. This depends on the starting values. For the analyses in this paper, the starting value for the label z_*i *_was 1 if gene *i*'s observed log ratio, minus the mean value for all genes and divided by the standard deviation of the values across all genes, was greater than 2 in absolute value, and 0 otherwise. This appeared to give good results.

The final label estimate for gene *i*, , is the posterior probability that it is differentially expressed, given the parameter estimates. The posterior probabilities do not need to be adjusted for multiple comparisons.

### Normalizations

There are two different types of experimental setup for which we will discuss normalization. The first is where the two different samples, say control and treatment, have each been labeled with a different color dye, say treatment with red (Cy5, R) and control with green (Cy3, G). In the second experimental setup, the treatment and control samples have replicates, with both control and treatment replicates being labeled with the same dye, say red (Cy5, R), and these are compared to a reference sample labeled with the other dye.

Two of the data sets analyzed in this paper, the HIV and the Like-like datasets, are of the first type of setup. The other data set analyzed in this paper is the Apo AI mouse data [1] which is of the second type of setup, with pooled control mRNA used as its reference sample. Since there are slightly different normalizations and quantities of interest used for analysis in these two cases, we will discuss them separately below, referring to the first experiment type as the log ratio experiment (since the log ratios are the quantities of interest), and to the second as the log ratio difference experiment (since the differences of log ratios between control and treatment samples are the quantities of interest).

#### Normalizations for the log ratio cDNA experiment

The main problem in applying the Normal-Uniform mixture model is that the data need to be normalized in order for this model to be appropriate. In the basic type of cDNA experiment, the log ratio of expressions in the two samples is the quantity of interest. There are dye and other effects that add a bias, making the mean of the non-differentially expressed log ratios non-zero (see the Like-like example in the Results section). Also, the variance of the log ratios depends on the log of the total intensity, where the total intensity is defined as the product of the red and green intensities. We need to ensure that any normalization does not "pull in" the differentially expressed genes.

#### Single slide normalizations

The normalization of single slide log ratios is a two-step process. In the first step, the observed log ratios are regressed nonparametrically on the log intensities, using the lowess regression smoother [18], and the fitted value is subtracted from the observed log ratios. In our implementation, a modification, the loess smoother [19], is used in place of lowess. Specifically,



where *R *and *G *are the intensities in the red and green channels, and *c*(log(*RG*)) is the fitted value from loess regression of log(*R*/*G*) on log(*RG*), a situation we denote by .

We got good results with a loess span in the range 60% to 80%. This generally did a good job of normalizing the mean but not the spread.

The spread depends on the log intensity, log(*RG*), and we estimate a running mean absolute deviation by loess regression of the absolute mean-normalized log ratio on the log intensity. We then divide the mean-normalized log ratio by the loess-estimated mean absolute deviation in order to get our final estimate,



where . We got good results with a span between 10% and 20%. As can be seen from the figures in the Results section, this does a good job of making the log ratios for non-differentially expressed genes approximately normal and homoscedastic.

#### Multiple slide normalizations with dye swap

In dye swap experiments, there is an even number of replicates and they are divided into two groups with equal numbers of replicates. In the second group of replicates, the assignment of dyes to samples is the reverse of that in the first group. Log ratios in this case are taken with the different samples as numerator and denominator (since the assigned dyes will be different for the two groups and averaging must be done over the same ratio of samples not the same ratio of dyes). In that case, mean normalization is unnecessary, although normalization of the variance is still required. This is because we take the average of the log ratios across replicates, ensuring that the dye effect cancels out.

#### Multiple slide normalizations without dye swap

Here we take the average of log ratios and log intensities across replicates for each of the genes and apply the mean lowess normalization, given by equation (4), with average ratios and intensities in place of the single replicate log ratios and intensities.

The variance normalization is not the same for multiple replicate slides as for a single slide. Because the average log ratios are not robust to outliers, even after mean normalization, we carry out a normalization based on variation across replicates rather than on variation depending on intensities, to downweight the influence of outlying observations. If the empirical standard deviation of the log ratios across replicates is greater than the absolute mean-normalized average log ratio for a gene, we divide its mean-normalized average log ratio by its standard deviation. If the empirical standard deviation of the log ratios across replicates is small, defined as smaller than the absolute mean-normalized average log ratio, we divide instead by a constant. The constant is chosen to be a high percentile (we use the 99th) of the distribution of the standard deviations of genes for which the absolute mean-normalized average log ratio is greater than the standard deviation. This avoids a gene being declared differentially expressed just because its empirical across-replicate standard deviation is small, as can easily happen by chance when there are few replicates. Thus the mean- and variance-normalized log ratio for a given gene is:



where *m *is the number of replicates, *q*_*j *_is the mean-normalized log ratio of replicate *j*, *s *is the standard deviation of log ratios across replicates, and *k *is the chosen percentile of the distribution of standard deviations of genes whose absolute mean-normalized average log ratio is greater than their standard deviation.

#### Normalizations for the log ratio difference cDNA experiment

Here the quantity of interest is the difference in log ratios between control and treatment replicates. We define





where *n*_*treatment *_is the number of treatment replicates, *n*_*control *_is the number of control replicates, *n *= *n*_*treatment *_+ *n*_*control*_, *q*_*treatment*,*i *_is the log ratio of treatment replicate *i *and *q*_*control*,*j *_is the log ratio of control replicate *j*. With these definitions we give the multiple-replicates normalizations, defined analogously to those in the log ratio type experiment.

#### Multiple slide normalizations

We again use loess to allow dependence of the mean normalization of M on A in the following way:

*M*_*norm *_= *M *- *c*(*A*)     (9)

where *c*(*A*) = *loess*(*M *~ *A*), with the recommended span for the loess smoother being between 60% and 80%.

For the variance normalization we again use the information about the variance contained in the replicates to get a robust estimator of the overall variance. We calculate the variance of log ratios across the *n*_*control *_replicates in the control dataset and call this *V*_*control*_. Similarly we calculate the variance of log ratios across the *n*_*treatment *_replicates in the treatment dataset and call this *V*_*treatment*_. Our estimate for the standard deviation s, in M for each gene is given by



We then develop the variance normalization similarly to the previous log ratio type experiment case. The variance normalization is given by



### Summary of model and normalizations for different experiments

A summary of the quantities of interest (used in the normalizations and normal uniform mixture model) and the normalizations is given in Table 7. An R package called nudge to implement the different normalizations and fit the model in this paper will be made available soon at [9].

**Table 7:** Summary of Methods

Type of Experiment	Multiple Replicates?	Dye Swap?	Quantity of Interest	Mean Normalization	Variance Normalization
Sample 1 = Red, Sample 2 = Green	No	NA		Equation (4)	Equation (5)
Sample 1 = Red, Sample 2 = Green	Yes	No		Equation (4)	Equation (6)
Sample = Red, Sample = Green Sample = Green, Sample = Red	Yes	Yes		NA	Equation (6)
Sample 1 & 2 = Red, Reference = Green	Yes	NA	M, Equation (7)	Equation (9)	Equation (11)

### Methods for comparison with NUDGE

We now give brief descriptions of the methods for finding differentially expressed genes that will be used for comparison with NUDGE in the datasets examined in the Results section.

#### Rule of two

This simple but popular method, mentioned in [3], involves examining the ratios or average ratios of the two channels for each gene, and calling those genes with a ratio or average ratio greater than two or less than half, differentially expressed. It requires some initial normalization and its performance can depend on the normalization.

#### t test and adjusted t test

One of the most obvious first approaches to try for this problem is the classical *t *test, as used, for example, in [20]. A simple normalization consisting of centering the mean of the log ratios within each replicate is often used in this case. One needs to be able to estimate the standard deviations as well.

Because of the large number of tests being run (thousands in the usual cDNA experiment setup), the standard *t *test needs to be modified to account for the multiple testing. Traditionally the most popular adjustment has been the Bonferroni correction, as mentioned in [21]. For the Bonferroni correction with *N *genes/tests and significance level *α*, we instead call each test significant only if it is significant at the  level, controlling for the probability of one or more false positives.

#### EBarrays

This follows a hierarchical Bayes approach for modeling the gene expression levels as detailed in [22]. As in our approach, the data are assumed to be generated by a two-component mixture model, one component for differentially expressed and the other for non-differentially expressed genes, each with their own distribution. The parameters specifying these distributions are estimated from the data, whence the name Empirical Bayes.

Results in this framework are given for two different parametric models in [22]. In the first model, the observed intensities for the replicates in each channel are assumed to be independently generated from a gamma distribution with a channel-specific scale parameter. The scale parameters are, in turn, assumed to have an inverse gamma distribution, whose parameters are estimated from the whole dataset. In the second model, the log ratios are assumed to be normally distributed, with gene-specific means that are themselves normally distributed. To normalize, the authors divided the log ratio for a given gene and replicate by the average log ratio across genes for that replicate.

#### Significance analysis of microarrays (SAM) [4]

The statistic used to test for differential expression is a regularized *t *statistic, i.e. the mean value divided by the sum of the standard deviation and a constant. SAM controls the False Discovery Rate (FDR), i.e. the number of genes declared to be differentially expressed that are not in truth differentially expressed.

A rejection region is fixed and SAM uses a permutation analysis to estimate the FDR. The user then decides on an acceptable rejection region based on their preferences for FDR.
